# Objective Signal Analysis for Investigating Feasibility of Active Noise Cancellation in Hearing Screening

**DOI:** 10.3390/s22197329

**Published:** 2022-09-27

**Authors:** Hsiu-Lien Cheng, Ji-Yan Han, Wei-Zhong Zheng, Yen-Fu Cheng, Yuan-Chia Chu, Chia-Mei Lin, Ming-Chang Chiang, Wen-Huei Liao, Ying-Hui Lai

**Affiliations:** 1 Department of Biomedical Engineering, National Yang Ming Chiao Tung University, Taipei 112304, Taiwan; 2 Department of Otolaryngology-Head and Neck Surgery, Taipei Veterans General Hospital, Taipei 11217, Taiwan; 3 Department of Medical Research, Taipei Veterans General Hospital, Taipei 11217, Taiwan; 4 Institute of Brain Science, National Yang Ming Chiao Tung University, Taipei 112304, Taiwan; 5 Department of Otolaryngology-Head and Neck Surgery, School of Medicine, National Yang Ming Chiao Tung University, Taipei 112304, Taiwan; 6 Information Management Office, Taipei Veterans General Hospital, Taipei 11217, Taiwan; 7 Big Data Center, Taipei Veterans General Hospital, Taipei 11217, Taiwan; 8 Department of Information Management, National Taipei University of Nursing and Health Sciences, Taipei 11219, Taiwan; 9 Medical Device Innovation & Translation Center, National Yang Ming Chiao Tung University, Taipei 112304, Taiwan

**Keywords:** Pearson correlation, active noise cancellation, hearing screening

## Abstract

With the development of active noise cancellation (ANC) technology, ANC has been used to mitigate the effects of environmental noise on audiometric results. However, objective evaluation methods supporting the accuracy of audiometry for ANC exposure to different levels of noise have not been reported. Accordingly, the audio characteristics of three different ANC headphone models were quantified under different noise conditions and the feasibility of ANC in noisy environments was investigated. Steady (pink noise) and non-steady noise (cafeteria babble noise) were used to simulate noisy environments. We compared the integrity of pure-tone signals obtained from three different ANC headphone models after processing under different noise scenarios and analyzed the degree of ANC signal correlation based on the Pearson correlation coefficient compared to pure-tone signals in quiet. The objective signal correlation results were compared with audiometric screening results to confirm the correspondence. Results revealed that ANC helped mitigate the effects of environmental noise on the measured signal and the combined ANC headset model retained the highest signal integrity. The degree of signal correlation was used as a confidence indicator for the accuracy of hearing screening in noise results. It was found that the ANC technique can be further improved for more complex noisy environments.

## 1. Introduction

Audiometry is one of the most common methods for hearing care and management, and mobile-phone-application-based self-testing methods [1,2,3] have been increasingly proposed over recent years. Although these methods have demonstrated good reliability in quiet environments [2,4,5,6,7], mobile-phone-application-based audiometry remains limited by the requirements for ambient noise control [8,9,10,11]. In particular, users often face increased false positives in hearing tests owing to the difficulty in controlling ambient noise. Accordingly, many methods have been proposed for mitigating this problem.

Noise cancellation methods include passive noise cancellation (PNC) [12] and active noise cancellation (ANC) [13]. PNC is primarily implemented using design methods such as those for headphones [14,15] and the characteristics of sound-absorbing materials [16,17]. Previous studies have shown that a PNC method can be effective in reducing high-frequency (>2000 Hz) components of the noise spectrum; however, the processing of low-frequency components requires further improvements. Compared to PNC, ANC has a different noise reduction effect on the noise band characteristics [18]. ANC estimates the ambient noise through an additional circuit and algorithm. Thereafter, an ANC circuit generates anti-noise signals with a magnitude equal to and a phase opposite to those of the ambient noise, thereby superimposing this anti-noise signal and primary noise to produce destructive interference for reducing noise interference. In view of this, ANC headphones are increasingly being investigated for audiometric applications [19,20,21,22]. For example, Bromwich et al. [20] used a head and torso simulator and the objective acoustic power measurement method to investigate the hearing threshold measurement benefits of ANC headphones for ambient noise. The results of the study showed that the ANC headphone technique could only detect ambient sound at a 40 decibels hearing level (dB HL) with confidence. Recently, Chang, Luo, Lo, and Tai [21] further investigated the abilities of recent ANC frameworks to mitigate ambient noise. The experimental results showed that current technologies have improved significantly; however, the processed signals still interfere with noise, especially low-frequency noise.

Although recent studies have gradually demonstrated that ANC headphone technologies can help users improve the accuracy of hearing screening in noisy conditions, there remains much room for improvement. For example, the effectiveness of ANC headphones is affected by the types of headphones (in-ear headphones, over-ear headphones), different ANC algorithms, and different noise and volume factors. More specifically, studies have shown that the anti-noise signal generated by active noise-canceling headphones is affected by ambient noise characteristics, and such characteristics may corrupt spectral component of the signal (e.g., the signal required for pure tone audiometry) [23,24]. In view of this, an analysis tool for quantifying the reliability of ANC headphone audiometry using signal analysis methods will help users understand the correlation of processed signals to original pure tones obtained from currently available mobile-phone-application-based audiometry software.

From the perspective of signal analysis, when the environmental noise leak into the ear canal can be reduced by ANC headphone processing, the accuracy of audiometric measurements is improved. This experiment investigated the impacts of ANC technology on audio fidelity from the perspective of signal analysis, and then investigated the correlations between processed signals under different application contexts (ANC headphone model, noise pattern, noise volume, and active noise cancellation status) referring to Pearson correlation coefficients. The degree of correlation serves as an accurate indicator of hearing screening under ANC to achieve 25 dB HL hearing screening results, similar to those derived in a sound-treated booth. Moreover, the scores could be used as a reference for the reliability of self-administered audiometry in the future. In other words, it is assumed that a higher correlation between the signal after ANC headphone processing and the original signal in quiet indicates that the ANC technology is good at overcoming the ambient noise and retains the integrity of the original signal. Based on this concept, a correlation analysis [25,26] is often used to quantify the distortion of the measured signal in the ear canal. In this study, we analyzed the spectral correlation between an ANC-processed signal and a target pure tone by calculating the correlation score based on the Pearson correlation coefficient and then investigated the linear association, magnitude, and direction between the two signals [27]. The participants underwent parametric listening tests. Finally, the feasibility of the objective analysis approach demonstrated in this study was further analyzed. A set of objective analysis results is proposed herein as an indicator to help users understand the reliability of audiometric screening results obtained under noisy environments.

## 2. Method

### 2.1. Study Equipment and Test Materials

**Figure 1:**
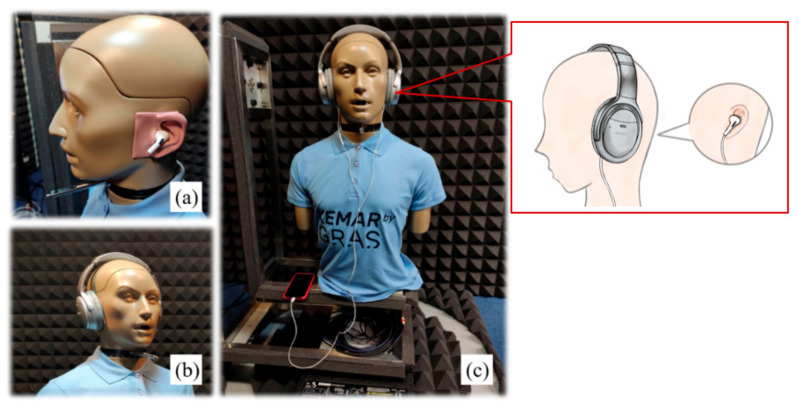
Two active noise cancellation (ANC) headphones constituting three ANC headphone models were used in the study: (**a**) in-ear, (**b**) over-ear, and (**c**) combined.

In this study, three commercial headphones were used (Apple AirPods Pro, Bose QuietComfort 35, and Apple EarPods). AirPods Pro and Bose QC35 contain ANC technology. According to ANC headphone-wearing, we classified AirPods Pro and Bose QC35 as in-ear and circumaural ANC headphone models. A combined ANC headphone model, which is composed of EarPods earphones covered by Bose QC35 ANC headphones, is designed for determining the effect of the composition on noise reduction. The constitution of three ANC headphone models for the experiment is illustrated in Figure 1. In addition, KEMAR (GRAS Sound & Vibration, Denmark) [28] manikins were used to record the audio from the three ANC headphone models. The pure tones used in this study were tested at the four frequencies (0.5 kHz, 1 kHz, 2 kHz, and 4 kHz) required for audiometric screening, and both steady (pink noise) and non-steady (cafeteria babble noise) noises [29] were selected as the background noise. Before the experiment, the sensitivity of the ear simulator and the electrical and acoustical measurement were calibrated by a pistonphone (G.R.A.S 42AP) generating a 94 dB SPL at 1000 Hz referring to the specification to attain the sensitivity of Kemar’s right and left ear. The output of three commercial headphones for 0.5–4 kHz were calibrated. First, an earphone was put on Kemar’s pinna, and the earphone and mobile phones were connected. Pure tones were generated via Android Studio software. The sound pressure was measured using Kemar’s ear microphone and was displayed in the CLIO electrical and acoustic system. The output level was compared and tuned using an audio programming code to meet the reference equivalent threshold sound pressure level (RETSPL re: ANSI S3.6) [30]. The calibrated output of three commercial headphones confirmed that an accurate 25 dB HL was required for audiometric testing.

### 2.2. Study Participants

Twelve adults were recruited for assessing the effectiveness of commercial ANC applied in 25 dB HL hearing screening under several noisy test conditions. The mean age of these subjects was 23.3 ± 1.7 years (22–27 years), and the 0.5–4 kHz pure tone average (PTA) values for the right and left ears were 7.3 ± 3.1 and 7.2 ± 3.8 dB HL, respectively. A total of 23 normal-hearing ears (including 11 right ears and 12 left ears) were considered in the study, following standard audiometric hearing as assessed by an experienced audiologist using the GSI AudioStar Pro audiometer with the RadioEar DD45 audiometric headset at Taipei Veterans General Hospital. The study protocol was approved by the Research Ethics Review Committee (2020-01-008BC) of Taipei Veterans General Hospital.

### 2.3. Proposed Objective Evaluation System

**Figure 2:**
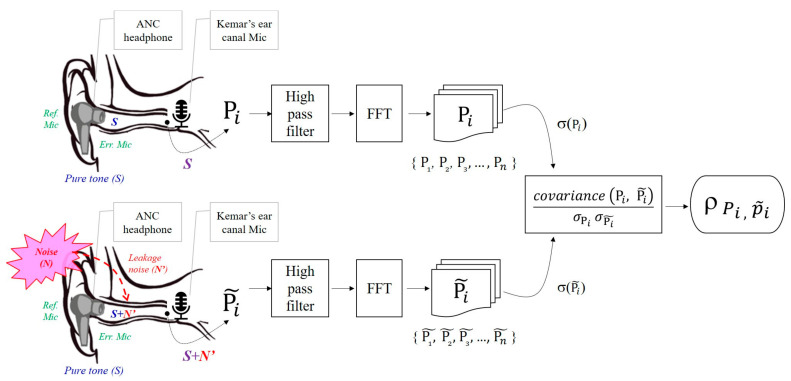
Flowchart for recording experimental acoustic signals and signal correlation calculations using the proposed signal analysis approach.

Figure 2 shows that an ANC headphone was worn in the ear canal and the objective signal analysis approach was demonstrated. Hybrid ANC comprises feedforward and feedback systems. The external microphone of the feedforward system receives ambient noise input as the reference microphone (Ref Mic) signal. The internal microphone of the feedback system receives the audios in the ear canal as an error microphone (Err Mic) signal. The leakage noise (N’) of ambient noise and pure tone (S) played from the earphone speaker were mixed in the ear canal and received by the error microphone and recorded by Kemar’s ear canal microphone. The Err Mic signal contributes to updating the weighting of the ANC algorithm. The audios recorded in the canal were used for further objective signal correlation coefficient analysis. The audio signals $P_{i}$ and ${\tilde{P}}_{i}$ are recorded in the mono channel at a sampling rate of 48,000 Hz, and then passing a 470 Hz high-pass filter for reducing electricity background noise to reduce the interference of the analyzed pure tones at 0.5 kHz. Both time domain signals $P_{i}$ and ${\tilde{P}}_{i}$ were aligned before a fast Fourier transform (FFT) analysis. Referring to the frame duration used in the speech signal process [31,32], a 32 ms frame length was selected for an FFT converted into its discrete-frequency components ($P_{i}$ and ${\tilde{P}}_{i}).$ The combination of 48 kHz sampling rate and frame length would be sufficiently implemented in analyzing the target frequency range of 0–4000 Hz that experiment focus on. The degree of correlation between the target pure tone signal ($p_{i}$) and the signal $\left({\tilde{p}}_{i}\right)$ after ANC headphone processed refers to the idea of Pearson’s product-moment correlation coefficient *r*. Pearson’s *r* denotes the magnitude and direction of the correlation between two variables and ranges from −1 to +1. If *r* is close to 1, the correlation is stronger, whereas if *r* is 0, the two variables are not correlated. In view of this, our study modified the idea of Pearson’s correlation coefficient to measure the degree of linear correlation between target pure tone ($P_{i}$) and ANC-processed signal (${\tilde{P}}_{i}$). The correlation coefficient (ρ) was calculated as the ratio of the covariance ($P_{i},{\tilde{P}}_{i})$ to the standard deviation ($\sigma_{P_{i}},\;\sigma_{{\tilde{P}}_{i}})$. A higher coefficient represented a more prominent target pure tone spectral component of ANC-processed signal. The correlation indicated the capacity of ANC headphones to save target signal integrity. Equation (1) was formulated for the correlation coefficient calculations, where index *i* represents the number of the FFT bin for each desired frequency of 0.5, 1, 2, and 4 kHz signal analysis and N is defined as the total frames. The relationship is expressed as follows:

(1) $$\rho_{i}\left(P,\tilde{P\;}\right)\%=\left(\frac{1}{N-1}\overset{N}{\underset{n=1}{\sum }}\left(\frac{P_{i,n}-u_{i,P}}{\sigma_{i,P}}\right)\left(\frac{{\tilde{P}}_{i,n}-u_{i,\tilde{P\;}}}{\sigma_{i,\tilde{P\;}}}\right)\right)*100\%$$

Finally, all the calculated results ($\rho_{i}$) were output to obtain the correlation score between the audio components of the active noise cancellation and were compared with those of the pure-audio component in a quiet environment.

### 2.4. Experiment Design

**Figure 3:**
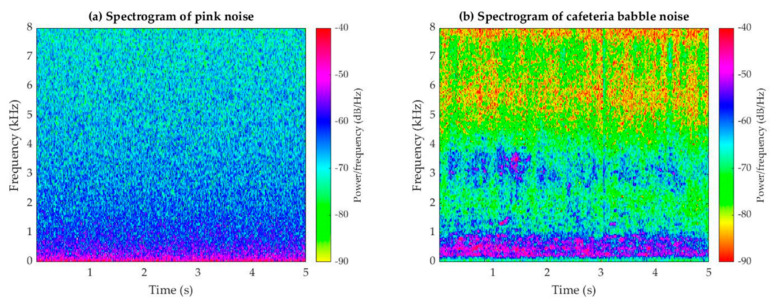
Audio spectra used in this study: (**a**) pink noise (steady noise) spectrum, and (**b**) cafeteria babble noise (non-steady noise) spectrum. The *x*- and *y*-axes denote time and frequency, respectively.

To prevent environmental noise from affecting the stability of the experiment, the experiment was conducted in a standard soundproof room by playing pink and cafeteria babble noise through speakers. The standard soundproof room is defined as the maximum permissible ambient noise level needed to fit the requirement of ANSI S3.1 standard, and the room noise level must meet the standard allowing a normal hearing patient to hear a tone presented as low as 0 dB HL. Additionally, there was only a single speaker used to play the background noise in a soundproof room in this study. The speaker position was based on the head of Kemar. The height of the speaker was 1 m from the ground and wall. The measured noise spectrum included third-octave band frequency analysis. To consider additional possible situations for users, five variables were considered: (1) ANC headphone model (in-ear, over-ear, and combined), (2) ANC function selection (ANC-ON/OFF), (3) pure-tone signal (0.5 kHz, 1 kHz, 2 kHz, and 4 kHz), (4) noise type (steady and unsteady), and (5) noise volume (50 dB, 60 dB, 70 dB, 80 dB, and 90 dB sound pressure levels (SPLs)). During the experiment, we recorded pure-tone signal characteristics in quiet and noisy environments, specifically, 36 times in a quiet environment (=four pure tones × three plays × three ANC models) and 360 times in a noisy environment (=four pure tones × three plays × three ANC models × two types of noise × five noise volumes). The experimental procedures are as follows: first, we placed an ANC model in Kemar’s pinna. The headphone was connected to the mobile phone application to play a pure tone in quiet. The pure tone was then recorded using Kemar’s microphone and saved as a quiet.wav file. Then, the noise was played through the speaker. The ANC function status was selected ON. The noise leak into the ear canal through the gap between the headphone and Kemar’s ear, and the leak noise mix up with desired pure tone as an ANC-processed signal, recorded via Kemar’s ear microphone and save as a noise.wav file. Both quiet and noise files were downloaded from the CLIO system. In the last procedure, our proposed signal analysis approach was applied for calculating the correlation score between ANC-processed signal and desired pure tone. The noise levels were calibrated as specified by ISO 8253-1 (International Organization for Standardization [ISO], 2010) [33] using the Kemar head position as the reference. The sound-level meter was set up at a height of 1 m from the floor and 1 m from the wall, and the noise spectrum was measured using a 1/3 octave band analysis covering a frequency range of 0.125–8 kHz. Next, the ambient noise level was adjusted on the sound-level meter according to the measurement results to meet the level required in the experimental design. Figure 3 shows the power spectra of the steady- and unsteady-state noises used in the experiment. After measuring the audio from different devices and settings in these conditions, we used the recorded sound in the quiet environment as the target signal ($P_{i})$ and the recorded signal in the noise scenario as ${\tilde{\;P}}_{i}$. Thereafter, the correlation coefficient analysis was performed using Equation (1).

## 3. Results and Discussions

Table 1 shows mean correlation scores of pure tone frequencies in ANC-processed signals under different noise volume for two noises, which derived from the results of Appendix A Table A1. Note that the Appendix A Table A1 presents all measurements of signal analysis and hearing screening respect to the ANC headphone model under noise pattern, noise volume, and pure-tone frequency. The signal analysis results at the pure-tone frequencies for the three ANC models are similar for the same background noise volume, regardless of noise types. The signal correlation scores are above 80% for both low- and high-frequency signals under the 50 dB SPL noise, with the highest correlation of 86% at 4 kHz, and the lowest correlation of 82% at 0.5 kHz. Under the 60 dB SPL noise, the signal correlation of the four pure-tone frequencies decreases to 60–66%, with the 4 kHz signal correlation score being the highest (66%) and the 0.5 kHz signal correlation score being the lowest (60%). At noise volumes above the 70 dB SPL, the signal correlation values of all the four pure tones are below 50%. In conclusion, the correlation scores of the ANC headphone for the measured signals between 0.5 and 4 kHz exhibit a gradual decrease as the noise volume is increased from 50 dB to 70 dB. In addition, the correlations of the measured signals at high frequencies are better than those at low frequencies; however, the correlation of the four pure-tone signals does not reach 70% when the noise volume exceeds the 60 dB SPL. At high noise levels, the correlation of the signals at low frequencies is better than that at high frequencies. Overall, the average signal correlation coefficients are 83%, 63%, 46%, 40%, and 35% as the noise level increases from the 50 dB SPL to the 90 dB SPL in 10 dB increments, respectively, and the overall signal correlation score decreases as the noise volume increases.

Table 2 depicts the signal average correlation scores of three ANC headphone models set at different noise types with different noise volume, which form the results of Appendix A Table A1. The signal analysis results from the three ANC headphone models decreased with different noise types and different noise volumes. The signal correlation values are 84%, 64%, 49%, 42%, and 35% for the 50–90 dB SPLs of the steady-state noise (in increments of 10 dB), respectively, and 82%, 62%, 44%, 38%, and 35% for the 50–90 dB SPLs of the unsteady-state noise, respectively. In the 50 dB SPL noise environment, the correlation scores of the three ANC models, regardless of the noise type, are maintained at over 82%. Moreover, only the signal correlation of the over-ear ANC model is slightly lower than 79%. The signal correlation score for the combined ANC model is higher than those for the in-ear and over-ear models. The signal correlation scores of the three ANC models are lower than 50% when the ambient noise is over the 70 dB SPL for both the steady and unsteady noise. Overall, the signal correlation score of the ANC models in stable noise environment is lower than that in non-stable noise. The experimental results confirm that the noise type has an impact on ANC. In other words, currently commercially available ANC technologies require an optimized noise-tracking method to improve the signal correlation when used in highly complex noise environments.

The abovementioned results show that the type of noise slightly affects the signal correlation. The signal correlation coefficients of the four pure-tone frequencies among three different ANC models at different noise levels, the signal correlation coefficients ρ of the 0.5 kHz, 1 kHz, 2 kHz, and 4 kHz pure tones for two different types of noise were further analyzed and compared with the pass rate (PR) of hearing screening with the corresponding ANC models (See Appendix A Table A1). The experimental results show that at 50 dB SPL of background noise, the signal correlation of the three different ANC models ranges between 75% and 93%, with the lowest correlation score (75%) at 1 kHz for the over-ear ANC headphone under unsteady-state noise and 87–100% for the corresponding 0.5–4 kHz audiometric PR. At the 60 dB SPL of noise, the signal correlation of the three different ANC models ranges between 54% and 77% at 0.5–4 kHz. The signal correlation score at 0.5 kHz is lower than that at other frequencies, with the lowest score at 0.5 kHz for the in-ear ANC model under unsteady-state noise (54%) and the highest score at 4 kHz for the combined ANC style under steady-state noise (77%). The corresponding PR for 0.5–4 kHz at the 60 dB SPL of noise is 59–100%, with the in-ear ANC model having the lowest PR at 1 kHz (59%) for the combined ANC model under unstable noise; the PRs for the over-ear and combined models at 2–4 kHz remains above 90%. At the 70 dB SPL noise level, the signal correlation obtained from three different ANC models is close to or below 50%, and only the combined ANC model has a high PR of 98–100% at 4 kHz under the 70 dB SPL. At the 90 dB SPL noise level, the audiometric PR at 0.5–1 kHz is 0% for all the three ANC models; however, the audiometric PR at 4 kHz is 46% for the combined ANC style. This is better than the performance of the in-ear and over-ear ANC headphones at the other pure-tone frequencies.

The three ANC models have high signal correlations for 0.5–4 kHz and higher audiometric screening PRs at the 50 dB SPL noise level. As the noise level increases, the signal correlations and audiometric screening PRs of the four pure tones for the three ANC models decrease. Overall, the three ANC models are comparable in terms of their abilities to maintain signal fidelity from 0.5 to 4 kHz; however, the combined ANC model is better than the in-ear or over-ear form factors in retaining the 4 kHz signal correlation and audiometric PR under high-noise environments. In brief, ANC technology can be implemented in audiometric testing in noisy environment.

**Figure 4:**
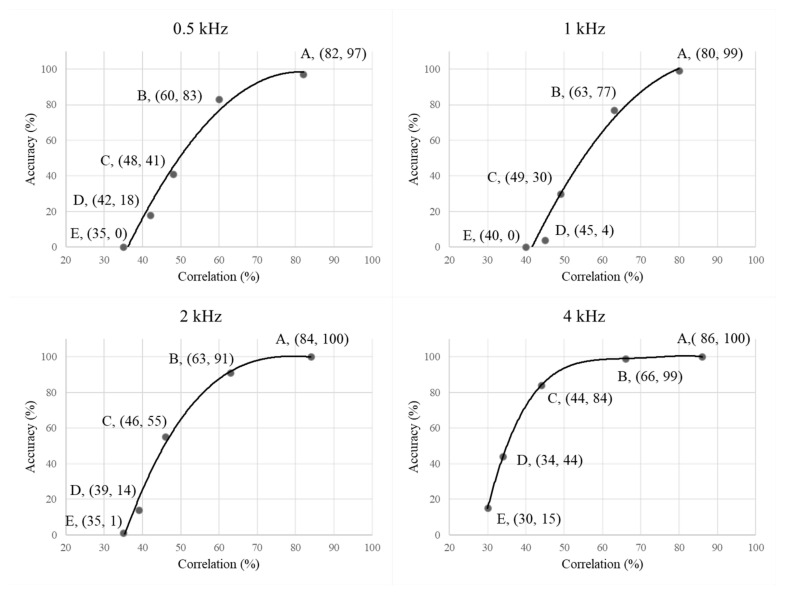
Plots between average signal correlation scores and accuracy of hearing screening for 0.5–4 kHz pure-tone frequencies under the ANC models in all test situations (3 ANC model ×2 ANC function status × 2 noise patterns × 5 noise volume × 4 pure tones × 2 set of acquisition), whose detailed results can be seen in Appendix A Table A1. Note that the results of correlation (%) were calculated from the Equation (1), and the results of accuracy (%) were obtained from the clinical hearing test.

Figure 4 illustrates the relationship between the results from the correlation calculation method (Equation (1)) and hearing screening. Specifically, the figure illustrates the relationship between the average signal correlation and audiometric accuracy for 0.5–4 kHz at the corresponding noise levels (point A–point E in Figure 4, i.e., A: 50 dB SPL, B: 60 dB SPL, C: 70 dB SPL, D: 80 dB SPL, and E: 90 dB SPL). The experimental results for point A indicate that ANC can maintain a high correlation (over 80%) for the four pure-tone signals in a 50 dB SPL noise environment; further, high consistency (over 97%) between the ANC audiometry and professional audiometry. The higher the signal correlation, the higher is the accuracy of the four pure-tone audiometry tests. At a comparable degree of signal correlation, the accuracy of the 0.5 kHz and 1 kHz audiometry is slightly lower than that at 2 kHz and 4 kHz, respectively, especially when the signal level does not reach 70%. There exists an evident difference between the accuracy of low-frequency audiometry and that of high-frequency audiometry, and when the signal correlation score is close to 50%, the accuracy of 0.5–2 kHz audiometry is unsatisfactory (at 60%). These results not only support the confidence in the accuracy of the audiometry results from the objective measurements of the signal fidelity but also allow for the limits of general ANC technology to reach the 60 dB SPL noise level. Accordingly, a goal of future developments pertaining to ANC applications for audiometry under noisy environments should be to improve ANC technologies for noise conditions above the 60 dB SPL.

This study introduced an objective analytical method for investigating the correlations between signal level and audiometric accuracy and developed an evaluation index to help users assess the reliability of self-administered audiometric results under different noise environments. Specifically, 0.5 kHz, 1 kHz, 2 kHz, and 4 kHz tones were used as audiometry signals, and the degrees between the processed and target signals for different ANC models were calculated using Pearson’s correlation coefficient (Equation (1)). Next, the relationship between the correlation scores and the accuracy of clinical audiometry was further investigated by comparing different signal correlation scores with the audiometric screening results. It was found that the three different ANC models used in this study provided signal correlation scores of over 80% (equivalent to the measured pure tone) under low noise levels (50 dB SPL) and achieved over 97% agreement with the clinical audiometry results (Figure 4). In other words, when the correlation calculated using the objective measurement index proposed herein exceeds 80%, users can achieve a measurement accuracy of over 97% under noisy environments, with the help of ANC technology. This also indicates that the three different ANC models can effectively eliminate ambient noise and maintain the fidelity of the target signal under low-noise-level conditions [34,35]. However, the signal correlation of the four frequencies used in the audiometry was found to decrease as the background noise volume increased. For the same experimental conditions (same type and volume of background noise), the signal correlation scores at each 0.5–4 kHz for the three different ANC models did not differ much. This is mainly because the active noise reduction headphones used in this study all employed similar digital-filtering techniques to execute the ANC algorithms [36]. In other words, under the same algorithmic framework, the correlations of the signals were similar across the different ANC models. This result also indicates that the proposed method will be applicable to most ANC headphone results under different frameworks in the future.

Although the three different ANC models have comparable noise cancellation capabilities, the signal correlation of the combined ANC model was found to be slightly better than those of the in-ear and over-ear models, likely owing to the double-blocking of the earmuffs [15,37] and the independent signal-source input. Consequently, the signal correlation of the combined ANC model at each noise level was higher than those of the other two models. In addition, the in-ear ANC headphones were the worst ANC model with respect to signal correlation. This is because they failed to fit snugly over the ear canal, and thus, ambient noise leaked into the ear canal [38]. Therefore, the combined ANC method was deemed the best among the three frameworks for future audiometric studies. In addition, we found that the signal correlation at 4 kHz was higher than that of the other pure-tone signals at noise levels up to the 60 dB SPL. When the noise level exceeded the 70 dB SPL, although the signal correlation for all the four frequencies was less than 50%, the signal correlations at 0.5 kHz and 1 kHz were slightly better than those at 2 kHz and 4 kHz under high noise levels. This is mainly because current ANC technologies possess good noise cancellation for low-frequency noise components (less than 1 kHz) [39] and the explanation could be the position of error microphone or signal processing challenge for ANC circuit. Limited distance between error microphone and eardrum or insufficient sampling rate make the high variant sound waves, such as high frequency signal an erroneous anti-noise estimation. Nonspecific characteristics of anti-noise signal reduce the destructive interference with primary noise for high frequencies, illustrating ANC work well at low frequencies [40,41]. Therefore, the benefits of ANC are more evident at these two frequencies. In other words, if ANC noise reduction technology continues to improve in the future, it will help users improve the accuracy of audiometry under highly challenging environments (e.g., above the 60 dB SPL), thereby enhancing hearing healthcare.

The present study found that the ANC function can improve the signal correlation and that the three types of commercially available ANC forms are generally effective. Once the ambient signal correlation falls below 60%, the 0.5–1 kHz audiometric accuracy is reduced to less than 83%, whereas the 2–4 kHz audiometric accuracy can still be maintained at over 90% with a signal correlation of 63%. This indicates that commercially available ANC headphones have limited functionality and must maintain high signal fidelity to enhance their audiometric accuracy at the low-frequency noise level of the 60 dB SPL. Therefore, there is still room for improvements in the optimization of ANC systems in the future, including improvements concerning the optimization of the ANC algorithm [42], digital computation time of the noise cancellation control system [40], load of the control system [24,42], and audio-quality perception of the ANC system [43].

Based on the analysis method for quantifying the signal correlation scores of the three ANC models proposed herein, the results confirm that the ANC technology can improve the signal correlation and that the three ANC models have equivalent abilities to retain the signal components at different SPLs of noise. However, the combined ANC model (Apple EarPods covered by BOSE QC 35 ANC headphone) maintained the best signal correlation. In addition, the higher the signal correlation, the higher was the accuracy of the ANC audiometry results. From the results of the present study, we believe that if the ANC system is separated from the playback signal system, it not only enhances the effectiveness of the ANC functions but also serves as a basis for quantifying the fidelity of the signal under noisy environments during audiometry. Moreover, it increases the confidence in the results of the ANC technology for audiometry. Furthermore, we found that general ANC technologies have a limited capability to handle noises above the 60 dB SPL, resulting in the degradation of low-frequency signal components. In the future, ANC systems can be optimally trained for 60 dB SPL noise to improve the fidelity of low-frequency signals, while enhancing the accuracy of low-frequency audiometry. The validation method for signal fidelity proposed herein not only quantifies signal variations under noisy environments but can also be used for the self-adjustment and management of over-the-counter (OTC) hearing aids [44]. In addition to other validation methods [45,46], this study can serve as a reference for managing the safety and quality of OTC devices.

## 4. Conclusions

The results of the present study demonstrated that the three different ANC headphone models provided good signal correlation scores at low noise levels (below the 60 dB SPL), with the combined ANC headphone model being the most effective in maintaining signal fidelity. In addition, the accuracy of the audiometric results obtained when the signal correlation reached 82% was found to exceed 97%. The signal components of commercially available ANC headphones with a 60 dB SPL of noise were found to be slightly insufficient, and their audiometry accuracy was also low. Thus, we suggest that the optimization of the ANC system algorithm and the use of deep learning to identify environmental noise characteristics can be employed to enhance the effectiveness of ANC and improve signal correlation, in order to afford a method for quantifying the environmental noise while self-administered hearing test.

## Figures and Tables

**Table 1:** Mean correlation scores of 0.5–4 kHz in ANC-processed signals under 50–90 dB SPL noises, where the detailed results of these average scores can refer to the Appendix A Table A1.

Noise Volume (dB SPL)	0.5 kHz	1 kHz	2 kHz	4 kHz	Average
50	82%	80%	84%	86%	83%
60	60%	63%	63%	66%	63%
70	48%	49%	46%	44%	46%
80	42%	45%	39%	34%	40%
90	35%	40%	35%	30%	35%

**Table 2:** Signal average correlation scores of the three ANC headphone models with respect to different noise pattern with different noise volume, where the detailed results of these average scores can refer to the Appendix A Table A1.

Noise Volume (dB SPL)	In-Ear		Over-Ear		Combined		Average	
	S	US	S	US	S	US	S	US
50	85%	83%	82%	79%	85%	84%	84%	82%
60	60%	58%	62%	62%	69%	66%	64%	62%
70	50%	41%	48%	44%	50%	47%	49%	44%
80	45%	34%	39%	38%	42%	41%	42%	38%
90	34%	33%	34%	35%	36%	37%	35%	35%

Note: S: steady-state noise, US: unsteady-state noise.

## Data Availability

Not applicable.

## Appendix A

**Table A1:** Signal correlation scores of ANC-processed signals and the relative pass rate of hearing screening under the application context for ANC headphone model, ANC function status (ANC-ON and ANC-OFF) at 500–4000 Hz under 50–90 dB SPL stable and unstable noisy condition.

Noise Volume (dB SPL)		In-Ear ANC Model															
		500 Hz				1000 Hz				2000 Hz				4000 Hz			
		ANC-ON		ANC-OFF		ANC-ON		ANC-OFF		ANC-ON		ANC-OFF		ANC-ON		ANC-OFF	
		ρ	PR	ρ	PR	ρ	PR	ρ	PR	ρ	PR	ρ	PR	ρ	PR	ρ	PR
Stable noise	50	85%	100%	84%	96%	86%	100%	85%	96%	89%	100%	83%	96%	86%	100%	85%	96%
	60	64%	96%	52%	87%	63%	83%	51%	57%	70%	78%	54%	78%	74%	96%	53%	91%
	70	56%	83%	47%	13%	50%	39%	47%	8%	57%	17%	52%	4%	48%	57%	41%	48%
	80	49%	61%	45%	0%	53%	9%	40%	0%	48%	4%	46%	0%	40%	13%	39%	0%
	90	38%	0%	28%	0%	44%	0%	23%	0%	38%	0%	35%	0%	33%	0%	31%	0%
Unstable noise	50	86%	87%	76%	87%	85%	96%	80%	96%	82%	100%	80%	100%	90%	100%	87%	100%
	60	73%	87%	35%	52%	75%	78%	59%	48%	67%	87%	42%	83%	76%	100%	37%	100%
	70	38%	61%	31%	0%	62%	35%	50%	0%	40%	43%	37%	13%	40%	74%	28%	78%
	80	29%	26%	25%	0%	59%	4%	49%	0%	33%	13%	28%	4%	28%	9%	24%	0%
	90	29%	0%	22%	0%	59%	0%	46%	0%	33%	0%	26%	0%	24%	0%	23%	0%
**Noise volume** **(dB SPL)**		**Over-ear ANC model**															
		**500 Hz**				**1000 Hz**				**2000 Hz**				**4000 Hz**			
		**ANC-ON**		**ANC-OFF**		**ANC-ON**		**ANC-OFF**		**ANC-ON**		**ANC-OFF**		**ANC-ON**		**ANC-OFF**	
		**ρ**	**PR**	**ρ**	**PR**	**ρ**	**PR**	**ρ**	**PR**	**ρ**	**PR**	**ρ**	**PR**	**ρ**	**PR**	**ρ**	**PR**
Stable noise	50	86%	100%	80%	100%	80%	100%	80%	100%	85%	100%	81%	100%	83%	100%	81%	100%
	60	67%	100%	65%	91%	63%	95%	59%	95%	63%	95%	60%	100%	64%	100%	57%	100%
	70	56%	91%	46%	14%	49%	64%	47%	27%	51%	67%	50%	62%	45%	95%	41%	95%
	80	41%	36%	41%	0%	40%	5%	36%	0%	48%	14%	40%	10%	35%	50%	32%	41%
	90	39%	0%	27%	0%	39%	0%	31%	0%	45%	5%	36%	0%	33%	5%	24%	5%
Unstable noise	50	85%	100%	76%	100%	75%	100%	74%	100%	81%	100%	79%	100%	80%	100%	80%	100%
	60	58%	100%	53%	41%	67%	91%	58%	73%	65%	95%	66%	86%	69%	100%	59%	100%
	70	52%	50%	44%	0%	47%	32%	41%	0%	44%	62%	39%	48%	45%	86%	37%	82%
	80	42%	0%	32%	0%	42%	0%	41%	0%	42%	33%	35%	14%	41%	41%	29%	32%
	90	41%	0%	28%	0%	43%	0%	31%	0%	42%	5%	34%	0%	39%	0%	25%	0%
**Noise volume** **(dB SPL)**		**Circumaural ANC model**															
		**500 Hz**				**1000 Hz**				**2000 Hz**				**4000 Hz**			
		**ANC-ON**		**ANC-OFF**		**ANC-ON**		**ANC-OFF**		**ANC-ON**		**ANC-OFF**		**ANC-ON**		**ANC-OFF**	
		**ρ**	**PR**	**ρ**	**PR**	**ρ**	**PR**	**ρ**	**PR**	**ρ**	**PR**	**ρ**	**PR**	**ρ**	**PR**	**ρ**	**PR**
Stable noise	50	86%	100%	84%	100%	82%	100%	74%	100%	89%	100%	88%	100%	91%	100%	87%	100%
	60	74%	100%	57%	83%	69%	100%	57%	91%	72%	100%	66%	100%	78%	100%	76%	100%
	70	59%	96%	41%	26%	59%	52%	43%	39%	51%	87%	41%	91%	61%	100%	47%	100%
	80	56%	39%	39%	9%	52%	17%	39%	4%	41%	30%	36%	17%	43%	96%	32%	96%
	90	53%	0%	34%	0%	46%	0%	31%	0%	32%	0%	29%	0%	37%	43%	28%	39%
Unstable noise	50	86%	100%	72%	96%	84%	100%	74%	100%	85%	100%	83%	100%	93%	100%	93%	100%
	60	66%	96%	59%	61%	67%	87%	66%	30%	67%	96%	57%	96%	79%	100%	67%	100%
	70	55%	52%	54%	4%	53%	48%	41%	22%	43%	87%	39%	74%	48%	100%	43%	96%
	80	51%	43%	49%	0%	51%	4%	41%	0%	37%	22%	34%	9%	36%	87%	30%	70%
	90	45%	0%	35%	0%	50%	0%	40%	0%	36%	0%	35%	0%	32%	52%	26%	39%

ρ: signal correlation score; PR: pass rate.
